# Static Magnetic Field Exposure Reproduces Cellular Effects of the Parkinson's Disease Drug Candidate ZM241385

**DOI:** 10.1371/journal.pone.0013883

**Published:** 2010-11-08

**Authors:** Zhiyun Wang, Pao-Lin Che, Jian Du, Barbara Ha, Kevin J. Yarema

**Affiliations:** Department of Biomedical Engineering, The Johns Hopkins University, Baltimore, Maryland, United States of America; University of Nebraska, United States of America

## Abstract

**Background:**

This study was inspired by coalescing evidence that magnetic therapy may be a viable treatment option for certain diseases. This premise is based on the ability of moderate strength fields (i.e., 0.1 to 1 Tesla) to alter the biophysical properties of lipid bilayers and in turn modulate cellular signaling pathways. In particular, previous results from our laboratory (Wang *et al*., BMC Genomics, 10, 356 (2009)) established that moderate strength static magnetic field (SMF) exposure altered cellular endpoints associated with neuronal function and differentiation. Building on this background, the current paper investigated SMF by focusing on the adenosine A_2A_ receptor (A_2A_R) in the PC12 rat adrenal pheochromocytoma cell line that displays metabolic features of Parkinson's disease (PD).

**Methodology and Principal Findings:**

SMF reproduced several responses elicited by ZM241385, a selective A_2A_R antagonist, in PC12 cells including altered calcium flux, increased ATP levels, reduced cAMP levels, reduced nitric oxide production, reduced p44/42 MAPK phosphorylation, inhibited proliferation, and reduced iron uptake. SMF also counteracted several PD-relevant endpoints exacerbated by A_2A_R agonist CGS21680 in a manner similar to ZM241385; these include reduction of increased expression of A_2A_R, reversal of altered calcium efflux, dampening of increased adenosine production, reduction of enhanced proliferation and associated p44/42 MAPK phosphorylation, and inhibition of neurite outgrowth.

**Conclusions and Significance:**

When measured against multiple endpoints, SMF elicited qualitatively similar responses as ZM241385, a PD drug candidate. Provided that the *in vitro* results presented in this paper apply *in vivo*, SMF holds promise as an intriguing non-invasive approach to treat PD and potentially other neurological disorders.

## Introduction

Parkinson's disease (PD) is an age-related disorder arising from the degeneration of dopaminergic nigrostriatal neurons of the basal ganglia resulting in dykinesia, tremor and rigidity. Current therapy – exemplified by the dopaminergic agent L-3,4-dihydroxy-phenylalanine (L-DOPA) – is restricted to symptomatic relief because agents capable of reversing or even effectively inhibiting neuronal degeneration have not yet been found. Compounding these limitations, L-DOPA therapy tends to lose effectiveness over time, L-DOPA-induced dyskinesias are a common complication of chronic dopaminergic therapy, and metabolites of this compound are neurotoxic [1]. The search for alternate, non-dopaminergic therapies to overcome these drawbacks has positioned adenosine A_2A_ receptor (A_2A_R) antagonists as an attractive option for improved treatment of PD [2], [3].

Despite the favorable features of A_2A_R antagonists, their pharmacological properties (e.g., poor oral availability and a lack of BBB permeability) constitute a barrier to clinical use. Consequently, alternative therapies including electromagnetic (EM) field exposure have been explored for PD. These efforts date back at least two decades when reports that high-frequency deep brain stimulation (DBS) could ablate certain aspects of neurological movement disorders were published [4]. Building on DBS, EM treatment modalities that fully penetrate the brain non-invasively have been pursued. For example, time invariant (i.e., static) magnetic fields of 1160 to 2600 gauss (0.116 to 0.260 T, similar to the field strength used in the current study) were shown to mimic the effect of caffeine, a nonspecific adenosine receptor antagonist that has inhibitory effects on neurons [5] and Sandyk and coworkers reported that magnetic fields ameliorated PD symptoms [6]. Interest in exploiting EM treatments for brain disorders continues today, exemplified by recent reports that EM radiation can reverse plaque formation in a murine model of Alzheimer's disease [7].

In light of two decades of investigation, the current study revisits the use of EMF exposure for PD by using moderate strength static magnetic fields (SMF) in the tenths of Tesla (thousands of Gauss) range where effects on biological molecules and physiological endpoints of potential therapeutic relevance have been unambiguously established. In particular, the current report builds on a genomics analysis of human embryoid body derived (hEBD) cells exposed to 0.23–0.28 T static magnetic fields that engaged signaling pathways related to neural function, broadly establishing relevance to PD [8]. More specifically, two facets of the study by Wang and coauthors [8] suggested relevance of SMF to PD. First, SMF exposure over short time periods increased IL-6 levels but suppressed IL-6 production over several days; similar responses – if they occur *in vivo* – could promote beneficial A_1_R activity over the short term [9], [10] and ameliorate the high levels of IL-6 found in the brains of Parkinson's patients over the longer term [11]. Second, software analysis of metabolic pathways showed that SMF impinged upon amino acid metabolism, suggesting that this stimulus could modulate aberrant amino acid metabolism associated with brain dysfunction.

In the current study, we investigated whether SMF could modulate PD-relevant endpoints in the PC12 rat adrenal pheochromocytoma cell line [12]. PC12 cells are widely used as an *in vitro* model to study PD [13], [14] because they possess intracellular substrates for dopamine (DA) synthesis, metabolism and transport and abundantly express adenosine A_2A_ receptors (e.g., A_2A_R) implicated in PD [15]–[20]. Using this model, we compared the effects of SMF with the A_2A_R-specific antagonist ZM241385 on PD-relevant parameters and found that SMF elicited similar responses against several endpoints. These results raise the intriguing possibility that this non-invasive stimulus could function as a substitute for small molecule A_2A_R antagonists under development as PD drugs.

## Results

### Exposure to SMF alters calcium flux in PC12 cells

**Figure 1:**
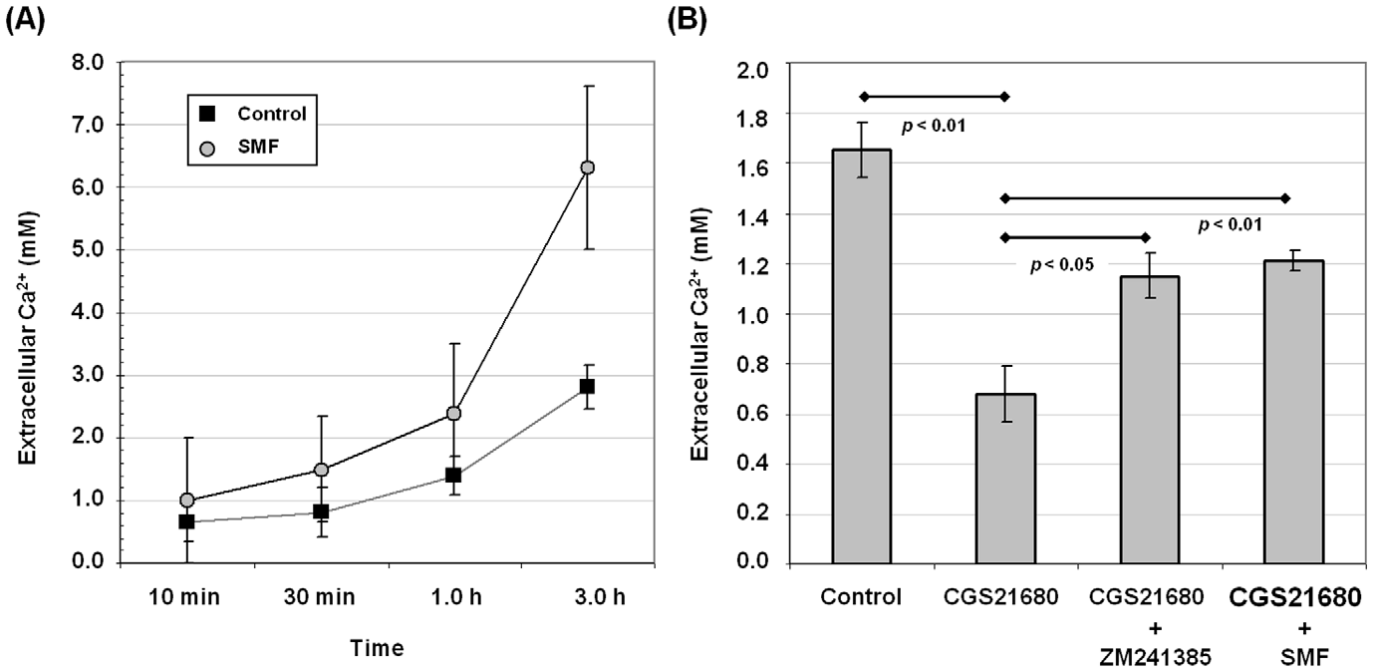
Calcium levels in PC12 cells exposed to SMF, the A_2A_R agonist CGS21680 or antagonist ZM241385. (**A**) Extracellular Ca^2+^ was measured for cells maintained in calcium-free medium increased for time points up to 3.0 h in response to SMF exposure; *p*<0.05 for n = 3 independent experiments. (**B**) In a separate experiment cells were evaluated at the three hour time point when the largest difference between SMF-treated and untreated cells occurred but before cell integrity was compromised from the assay conditions (e.g., from using Ca^2+^ and Mg^2+^ free D-PBS). Cells treated with 1.0 µM CGS21680 experienced decreased Ca^2+^ release compared to control cells while co-treatment of the cells with this agonist and either 1.0 µM ZM241385 or SMF attenuated the CGS21680-induced decrease (*p* values for each comparison are shown on the chart for n = 3 independent experiments).

Altered calcium flux is a well established cellular hallmark of exposure to SMF [21]; the first objective of the current study was to verify that this endpoint – previously observed in lymphocytes, HepG2, U937, HeLa, COS7, and hEBD lines [8], [21] – was affected by magnetic exposure in PC12 cells. As shown in Figure 1A, efflux of Ca^2+^ from SMF-treated cells, measured by the level of Ca^2+^ in the supernatant, diverged from untreated cells over a three hour period and, as described in our previous publication [8], reciprocal changes to intracellular Ca^2+^ levels occurred under these assay conditions (data not shown). A second objective was to verify that CGS21680, a selective adenosine A_2A_ receptor (A_2A_R) agonist that reproduces cellular responses that contribute to PD, inhibits calcium currents and related biological endpoints in PC12 cells in our assays as reported in other studies [15], [22]–[24]. As shown in Figure 1B, CGS21680 substantially inhibited Ca^2+^ efflux in PC12 cells, decreasing extracellular Ca^2+^ levels by ∼50% compared to untreated controls. Co-incubation of the CGS21680-treated cells with ZM241385, a potent, non-xanthine A_2A_R antagonist [25] under evaluation as a drug candidate for PD [2], [26] partially, but substantially, offset this inhibition. Importantly, foreshadowing subsequent endpoints investigated in this study, the ability of ZM241385 to counteract the effects of CGS21680 was reproduced by SMF.

### SMF exposure changes A_2A_R mRNA and protein levels

**Figure 2 pone-0013883-g002:**
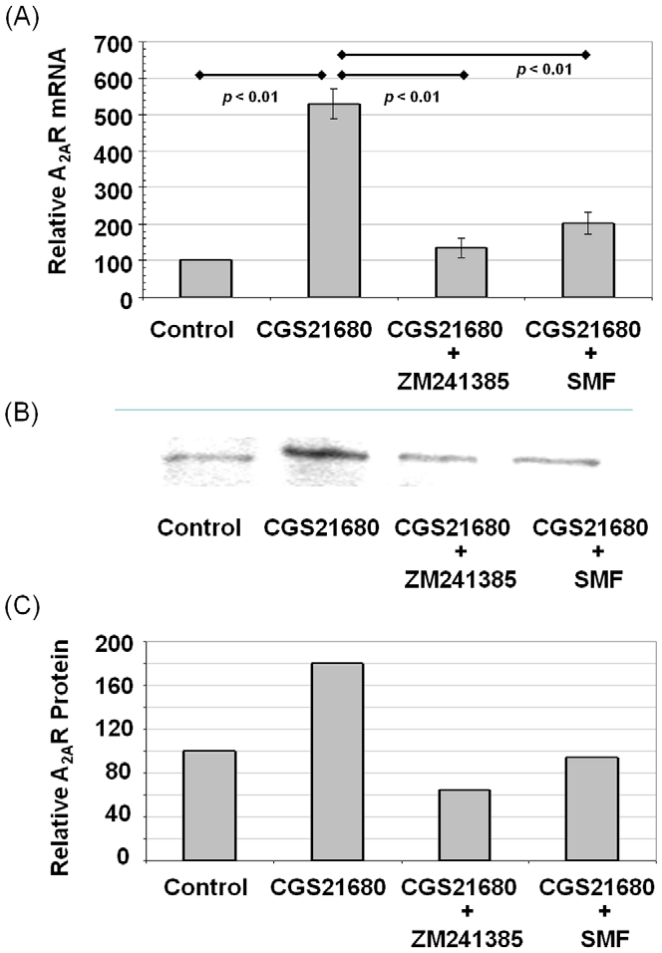
Effect of Ca^2+^ flux and adenosine activators and blockers on A_2A_R mRNA and protein levels in PC12 cells. (**A**) The A_2A_R agonist CGS21680 increased A_2A_R mRNA levels by over 5-fold while the antagonist ZM241385 as well as SMF decreased this agonist-enhanced A_2A_R transcription to close to control levels (*p* values for each comparison are shown on the chart for n≥3 independent experiments). (**B**) The A_2A_R agonist CGS21680 increased A_2A_R protein levels while the antagonist ZM241385 as well as SMF decreased A_2A_R in western blots; quantification of representative data is shown in (**C**); this experiment was repeated three times with similar results.

To investigate whether changes to Ca^2+^ flux observed at early time points in SMF-treated cells (Figure 1) impacted endpoints relevant to PD in PC12 cells over longer time periods, we measured A_2A_R mRNA and protein levels. In this experiment, the A_2A_R agonist CGS21680 dramatically up-regulated A_2A_R mRNA; this response was reversed by concurrent exposure to ZM241385 (Figure 2A). Consistent with the results shown in Figure 1 where ZM241385 was shown to reverse the impact of CGS21680 on calcium efflux, SMF was able to suppress the increased A_2A_R mRNA levels engendered by CGS21680. To confirm that the changes in mRNA expression extended to protein levels of A_2A_R, we used western blotting to compare A_2A_R in control and test cells and found that the highly increased amounts of A_2A_R mRNA in CGS21680-treated cells led to a similar (albeit quantitatively smaller) increase in A_2A_R protein levels. These increases in A_2A_R were reduced to roughly control levels by co-treatment with ZM241385 and SMF (Figure 2B & C).

### SMF mediated changes are consistent with L-type Ca^2+^channel modulators

**Figure 3:**
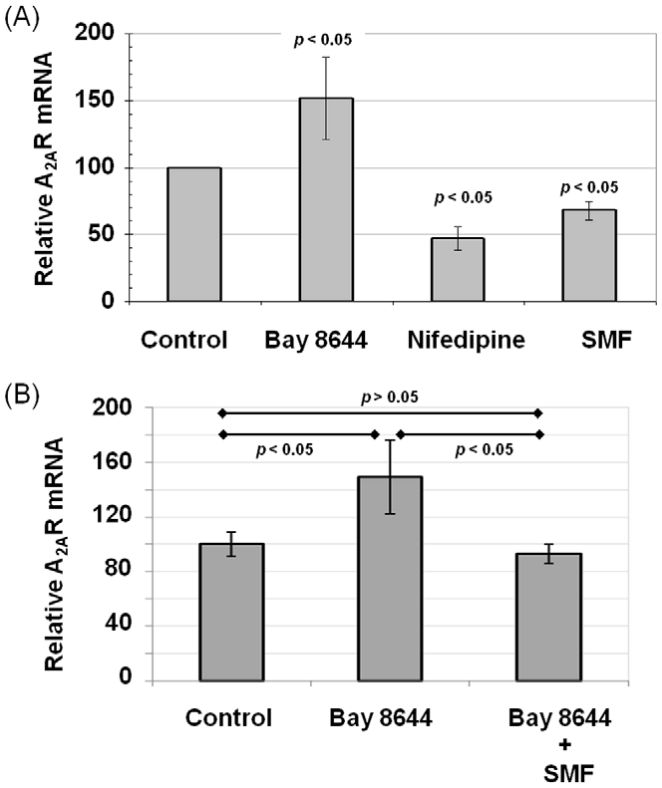
Effect of L-type Ca^2+^ channel activators and blockers on A_2A_R mRNA and protein levels in PC12 cells. (**A**) The L-type Ca^2+^ channel activator Bay K8644 increased A_2A_R mRNA levels in PC12 cells compared to untreated controls while the L-type Ca^2+^ blocker Nifedipine, as well as SMF exposure, decreased A_2A_R mRNA levels after 6.0 h of exposure (*p*<0.05 for each test condition compared to control for n≥3 independent experiments). (**B**) Increased A_2A_R mRNA resulting from exposure to Bay 8644 was reversed by concomitant exposure to SMF (*p* values are shown for n≥3 independent experiments).

To gain a better perspective whether long-lived changes (e.g., changes to gene expression, and endpoint previously observed for SMF in our studies [8]) could have been initiated through the proposed modulation of calcium channel activity by SMF, an independent method to alter Ca^2+^ flux was evaluated. Specifically, Bay K8644 (an L-type Ca^2+^ channel activator) and nifedipine, (an L-type Ca^2+^ channel blocker) were used to alter Ca^2+^ flux in PC12 cells and A_2A_R mRNA levels were again evaluated. In this experiment, Bay K8644 increased A_2A_R mRNA levels while nifedipine treatment decreased transcription (Figure 3A); in essence Bay K8644 reproduced the effects of agonist CGS21680 and nifedipine mimicked antagonist ZM241385 (as shown in Figure 2A). To further strengthen the correlation between L-type Ca^2+^ channels, calcium flux, and A_2A_R transcription, we demonstrated that the increased levels of A_2A_R mRNA found in Bay 8644 treated cells could be reduced to levels found in control cells by concomitant exposure to SMF (Figure 3B).

Overall, although detailed characterization of the intracellular flux of calcium in SMF-treated cells is beyond the scope of the current work (for example, real-time imaging methods that capture dynamic changes to organelle-specific calcium levels are not compatible with our SMF-treatment device) the experiments described in Figures 1 to [Fig pone-0013883-g002] 3 are consistent with a mechanism whereby SMF alters the biophysical properties of cellular membranes and embedded ion channels (see Discussion), thereby affecting Ca^2+^ flux in ways that mimic the A_2A_R antagonist ZM241385. Based on this foundation, and the knowledge that calcium functions as a second messenger in numerous signaling pathways and – in neural cells – contributes to the excitatory state [27], the remainder of this report describes several endpoints of relevance to PD that respond to SMF in a manner similar to ZM241385 in PC12 cells.

### SMF exposure modulates ATP and ADO levels

**Figure 4:**
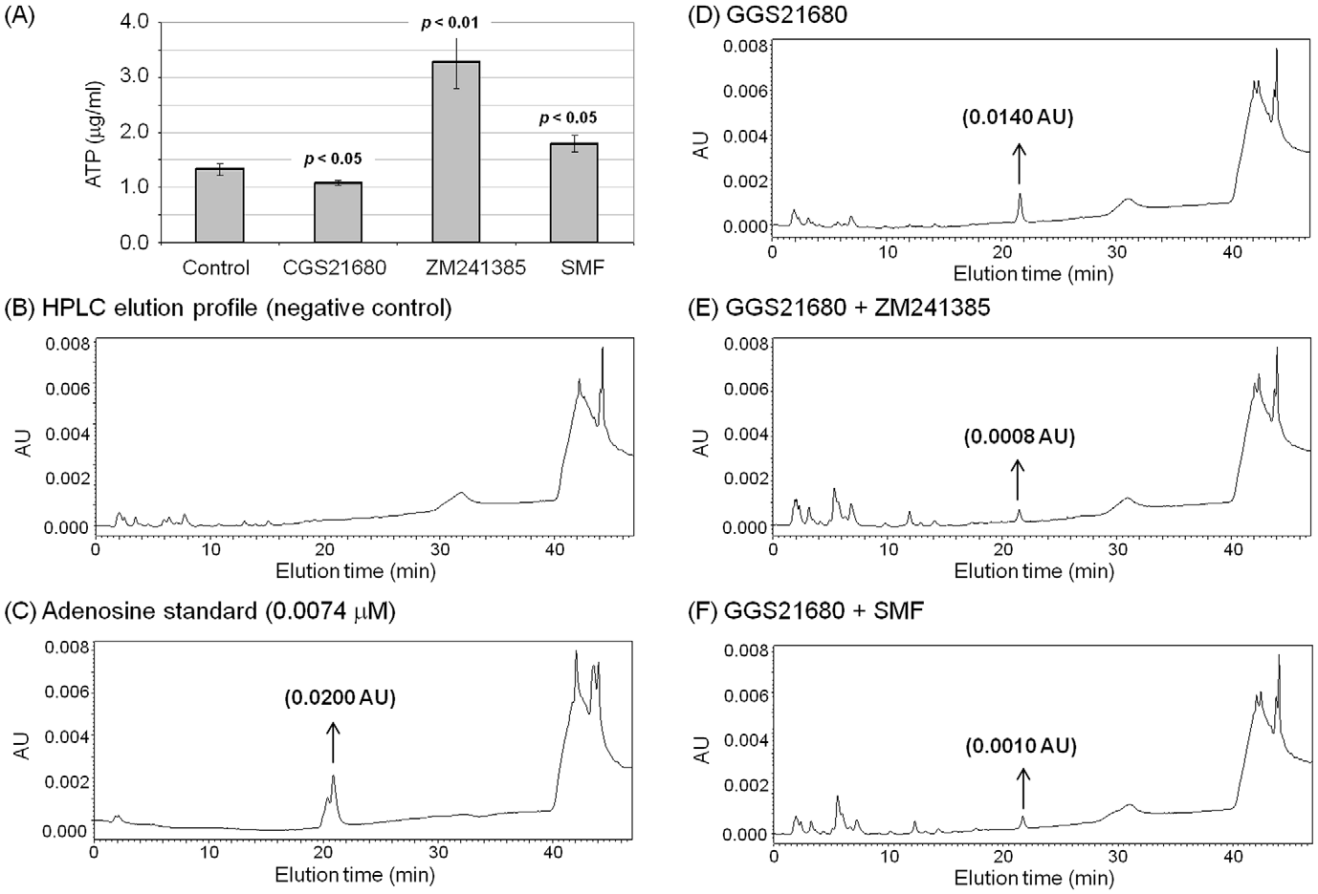
Cellular ATP and ADO levels in PC12 cells exposed to SMF, CGS21680 or ZM241385. (**A**) Cells were incubated with 1.0 µM CGS21680, ZM241385, or exposed to SMF for 6.0 h. The cells were harvested and an equal number from each sample were used to prepare extracts and to measure intracellular ATP levels (*p*<0.05 for n≥3 independent experiments for each treatment condition compared to untreated control cells; a similar trend was observed for 3.0 h, but not all data points were statistically significant). (**B**) – (**F**) After 3.0 h incubation in D-PBS, the extracellular fluid was collected from PC12 cells and analyzed by HPLC to detect and quantify ADO. (**B**) ADO was not detected in samples from untreated control cells (elution of authentic ADO is shown in (**C**)) but was observed in samples from cells treated with (**D**) 1.0 µM CGS21680, (**E**) 1.0 µM CGS21680 plus 1.0 µM ZM241385, or (**F**) 1.0 µM CGS21680 plus exposure to SMF in (**F**). The HPLC assays were repeated three times with similar results; representative data is shown.

Upon establishing that CGS21680, ZM241385, and SMF modulate Ca^2+^ ion channel flux and A_2A_R transcription in PC12 cells (Figures 1–3) we investigated whether the effects of these stimuli extended to modulation of adenosine (ADO) metabolism. Specifically, because calcium is linked to adenosine (ADO) levels that, together with cAMP, modulate A_2A_R activity in PC12 cells to reproduce cellular aberrations found in PD [17]–[20] we first measured cellular levels of adenosine triphosphate (ATP), which provide energy to activate the plasma membrane Ca^2+^ ATPase (PMCA) and also is an upstream source of ADO. ATP levels were moderately (but statistically significantly) lower in PC12 cells incubated with CGS21680 compared to untreated controls (Figure 4A), consistent with a shift to an ADO producer phenotype that occurs during hypoxia in this cell model of PD [28]. By contrast, ATP levels were higher in ZM241385 and SMF treated cells than in the untreated controls.

Known metabolic connections between ATP and the downstream metabolite ADO suggested that changes to ATP levels shown in Figure 4A would be reflected in changes to ADO, an important modulator of PD-related endpoints via adenosine receptors. Using an HPLC assay, we were unable to detect ADO release from untreated control cells (Figure 4B; an authentic ADO sample is shown in Figure 4C). By contrast, ADO release increased to readily detectable levels for CGS21680-treated cells (Figure 4D). The release of ADO from A_2A_R agonist-treated cells was attenuated by ∼50% by concurrent treatment with the small molecule antagonist ZM241385 (Figure 4E) as well as by SMF (Figure 4F).

### SMF exposure increases intracellular cAMP levels

**Figure 5:**
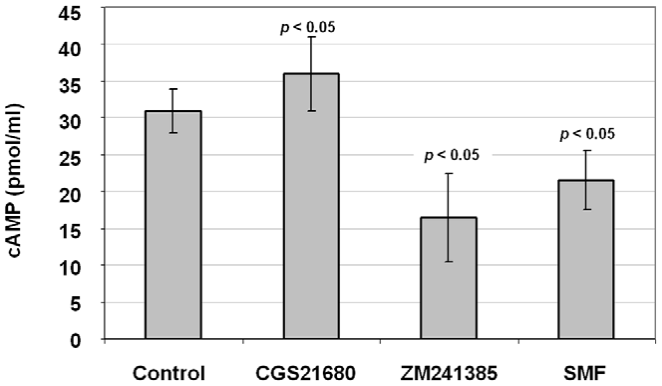
cAMP levels in SMF, CGS21680, and ZM241385 treated PC12 cells. Cells were exposed to each condition, harvested, lysed, and assayed for cAMP levels. Each test condition treatment condition varied from untreated control cells with *p*<0.05 for n = 3 independent experiments.

Levels of cAMP are another parameter relevant to PD that can be interrogated in PC12 cells; this ubiquitous second messenger is linked to Ca^2+^ through a complex sequence of events mediated by A_2A_R [29] and G_αs_ proteins [30]. To evaluate connections between cAMP and A_2A_R in our experiments, we analyzed cAMP levels in agonist (CGS21680) and antagonist (ZM241385) treated cells and found a modest increase in the former and a more substantial decrease in the latter (Figure 5). In these experiments SMF decreased cAMP levels, again showing that magnetic exposure can functionally reproduce the cellular effects of an A_2A_R antagonist.

### SMF, like A_2A_R antagonists, inhibits nitric oxide production in PC12 cells

**Figure 6:**
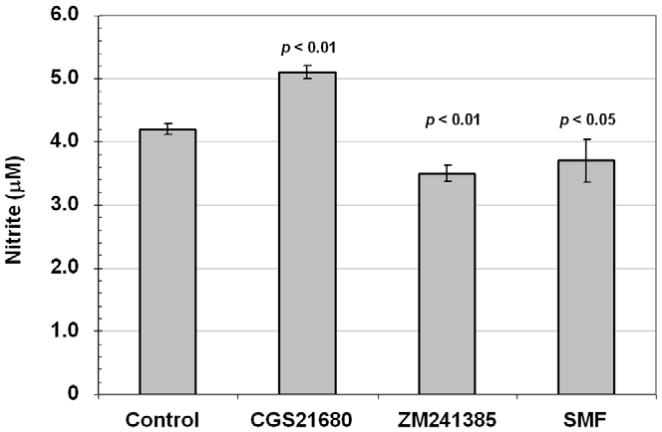
Effect of CGS21680, ZM241385, and SMF on nitrite levels in PC12 cells. Levels of nitrite were measured after 24 h of incubation with 1.0 µM of the A_2A_R agonist (CGS21680) or antagonist (ZM241385) or after exposure to SMF; *p* values are shown in comparison with untreated control cells for n = 3 independent experiments.

Nitric oxide (NO) is a molecular mediator of many physiological processes, including mechanisms that contribute to neurological disorders such as Alzheimer's disease and PD [31]. Therefore, because of reported connections between Ca^2+^, cAMP, and NO ([29]), we measured nitrite concentrations (nitrate is formed by the spontaneous oxidation of NO under physiological conditions) in PC12 cells. Nitrite levels increased in cells incubated with agonist (CGS21680) after 24 h of exposure while they decreased in antagonist (ZM2412385) treated cells (Figure 6). Consistent with results reported above for other PD-related endpoints, SMF reduced nitrite levels, once again demonstrating that magnetic exposure can mimic responses elicited by an A_2A_R antagonist.

### SMF impinges upon MAPK pathways and impacts PC12 cell proliferation

**Figure 7:**
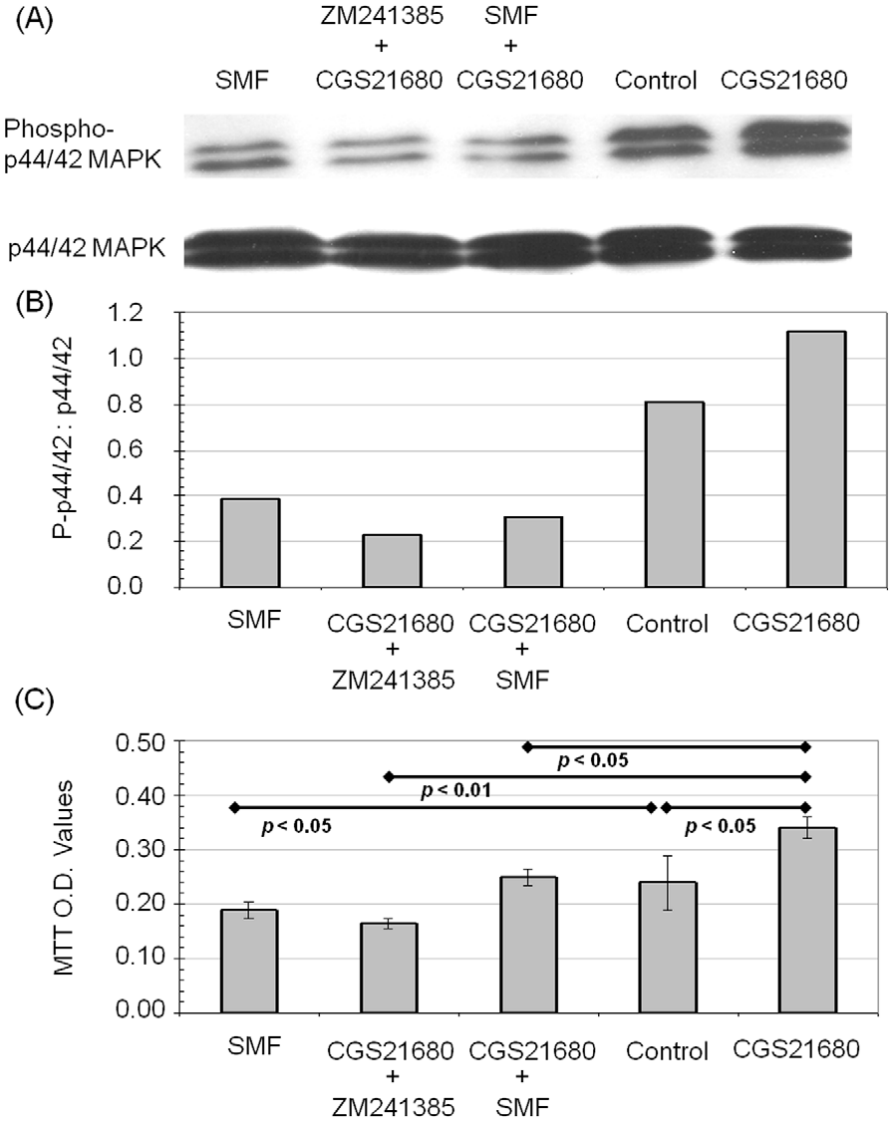
Effect of CGS21680, ZM241385, and SMF on p44/42 MAPK phosphorylation and proliferation in PC12 cells. (**A**) Western blots show the phosphorylation of p44/42 MAPK after exposure to SMF or 1.0 µM CGS21680 for 48 h, or after pretreatment with 1.0 µM ZM241385 or SMF for 30 min followed by the addition of 1.0 µM CGS21680 and an additional 48 h of incubation (total p44/42 MAPK is also shown); quantification by densitometry for a representative experiment (of n = 3 independent experiments) is shown in (**B**). (**C**) Proliferation of PC12 cells grown under the conditions indicated in (A), but for three days instead of 48 h, are given as measured by the MTT assay (*p* values for the various comparisons indicated on the figure are for n≥3 independent experiments).

Stimulation of PC12 cells with CGS21680 increases the phosphorylation of p44/42 MAPK (Erk1/2) via cAMP-mediated signaling [19], [32]. This prior observation, together with known links between NO production and phosphorylation of p44/42 MAPK [29], prompted us to test whether SMF and the A_2A_R modulators CGS21680 and ZM241385 also affected p44/42 MAPK. Accordingly, we first investigated whether CGS21680 increased the phosphorylation of p44/42 MAPK and observed an increase by Western blot analysis after 30 min of exposure (Figure 7A & B) that was consistent with enhanced proliferation observed in the agonist-treated cells (Figure 7C). By contrast, pretreatment of the cells with the ZM241385 or co-treatment with SMF reversed CGS21680-induced p44/42 MAPK phosphorylation resulting in levels lower than observed in untreated control cells (Figure 7A & B); the accelerated proliferation observed in CGS21680 treated cells also was not seen under these condition (Figure 7C). In these experiments, SMF by itself also reduced levels of phospho-p44/42 MAPK and proliferation.

### SMF inhibits neurite outgrowth in PC12 cells

**Figure 8:**
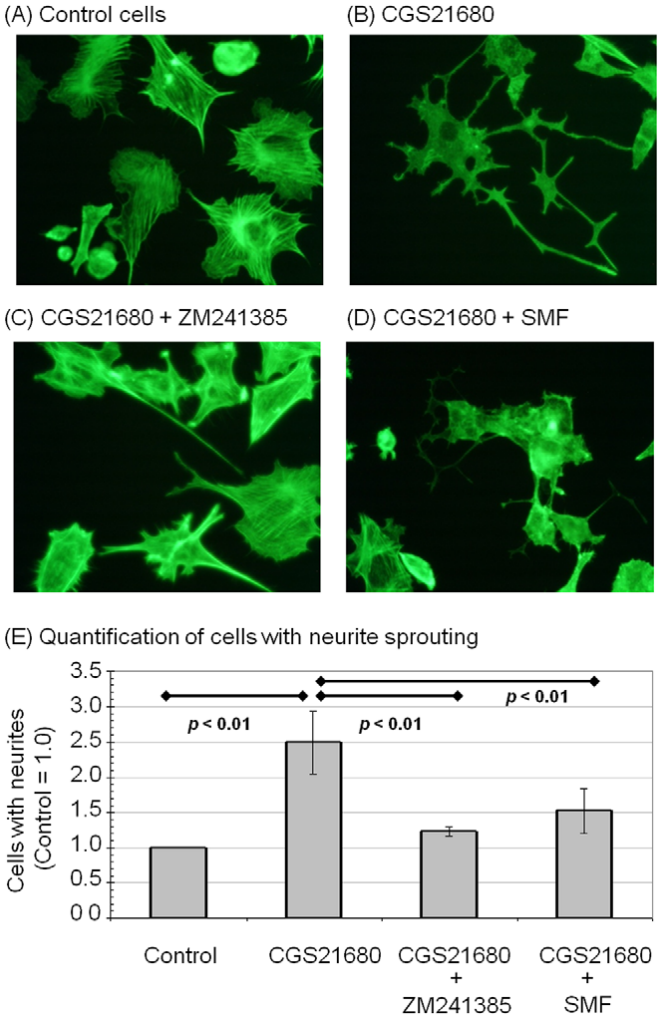
Effect of CGS21680, ZM241385, and SMF on neurite sprouting in PC12 cells. Representative images of PC12 cells that were (**A**) untreated or treated with (**B**) CGS21680 (1.0 µM), (**C**) ZM241385 (1.0 µM), or (**D**) SMF for three days are shown. (**E**) Neurite outgrowth was quantified by counting the number of cells exhibiting neurites that were 1.5 times longer than the diameter of the cell and the proportion of cells with neurites was expressed as a percentage of the total number of cells; data is shown from counting at least 100 cells from each of five fields selected at random.

The reduced proliferation of PC12 cells exposed to SMF could result from several underlying causes including the onset of apoptosis. Magnetic fields, however, have been reported to be anti-apoptotic [33] and the SMF conditions used in this report have previously been shown to not have a negative impact on cell viability [8]. Another possibility, supported by our previous work where human embryonic cells gained expression of pre-oligodendrocyte markers upon SMF exposure [8], was that the reduced proliferation we observed was a consequence of differentiation. To assess this possibility, changes to cell fate were monitored by measuring neurite outgrowth, which has been linked directly to A_2A_R (e.g., during hypoxia [34]) as well as indirectly (e.g., through cAMP-mediated crosstalk between the MAPK pathway and A_2A_R during exposure to the bacterial nucleoside N6-methyldeoxyadenosine [35]). In these experiments it was necessary to treat the PC12 for three days with CGS21680 to enhance neurite sprouting [36]; CGS21680 caused PC12 cells to flatten and to sprout extended long processes indicative of neurite outgrowth to a much greater extent than untreated controls (Figure 8A & B). ZM241385 counteracted the A_2A_R agonist-induced increase in neurite outgrowth (Figure 8C) and exposure of the CGS21680-treated cells to SMF had the same effect (Figure 8D).

### SMF inhibits iron uptake in PC12 cells

**Figure 9:**
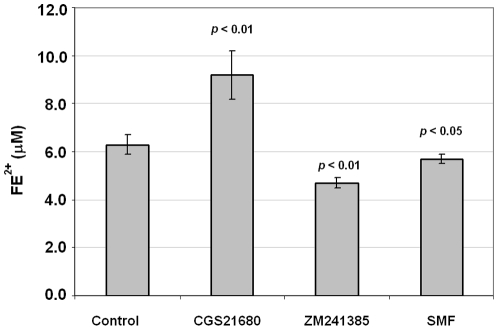
Effect of CGS21680, ZM241385, and SMF on iron uptake in PC12 cells. Intracellular iron was quantified using a colorimetric assay one hour after the addition of 50 µM FeSO_4_ to the medium (*p* values are shown comparing each test condition to controls for n≥3 independent experiments).

Iron uptake, which can occur via a Ca^2+^ activated non-transferrin bound iron (NTBI) mechanism in PC12 cells [37]–[40], is associated with several neurodegenerative diseases including PD and Alzheimer's [41] (in PD, oxidative stress hypothesis leads to increased iron concentration in the substantia nigra that induces progressive dopaminergic neuronal degeneration secondary to a high production of hydroxyl radicals by Fenton reaction [42]). Moreover, iron uptake varies between non-differentiated and NGF-induced differentiated PC12 cells [41]. These two factors – changes in Ca^2+^ flux (Figure 1) and indications of differentiation (i.e., neurite sprouting, Figure 8) – prompted us to investigate iron uptake in PC12 cells treated with CGS21680, ZM241385, or SMF. Exposure of PC12 cells to concentrations (50 µM) of free divalent iron (FeSO_4_) that ultimately lead to cell death showed that CGS21680 significantly enhanced iron intake at early time points (i.e., when the cells were still viable) whereas ZM241385 or SMF exposure inhibited agonist-promoted uptake (Figure 9). In essence, SMF decreased the bioavailability of Fe^2+^ thereby functioning in a manner similar to neuroprotective iron chelating drugs [41].

## Discussion

Numerous drawbacks with L-DOPA based therapy for PD (as reviewed elsewhere, [1], [43]) have led to intense efforts to develop alternative treatments. One direction has been guided by epidemiological evidence that heavy coffee drinkers have a lower incidence of PD [44] with the benefits of coffee presumably resulting from caffeine's interactions with adenosine receptors [45]. Consistent with this hypothesis, early clinical tests showed that non-specific adenosine receptor-antagonist theophylline provided significant benefits for PD patients [46]. Subsequent investigations that established that antagonistic interactions exist between A_2A_R and dopamine D2 receptors spurred the search for A_2A_R-specific antagonists [47] such as KW-6002, a compound that showed therapeutic value in MPTP-treated marmosets an animal model of PD [48]. In the past several years, highly selective A_2A_R antagonists – such as ZW241385 used in the current study – have been developed.

In this report we combine the emergence of A_2A_R as a target for PD drug development with the growing realization that magnetic exposure legitimately modulates physiological processes *in vivo* in ways that may be therapeutically beneficial [49]–[52] (overall, more than 40 randomized controlled trials of magnetic therapy for more than 30 clinical indications have been reported [53]) to show that SMF exposure reproduces the effects of A_2A_R antagonists over a gamut of PD-relevant endpoints in PC12 cells. More specifically the current experiments demonstrate that SMF can reproduce the effects of A_2A_R antagonist ZM241385 in PC12 cells or, in cases where an appropriate response could not be observed in naïve cells (e.g., ADO release (Figure 4) or neurite sprouting (Figure 8)), SMF can counteract responses induced or exacerbated by the A_2A_R agonist CGS21680.

**Figure 10:**
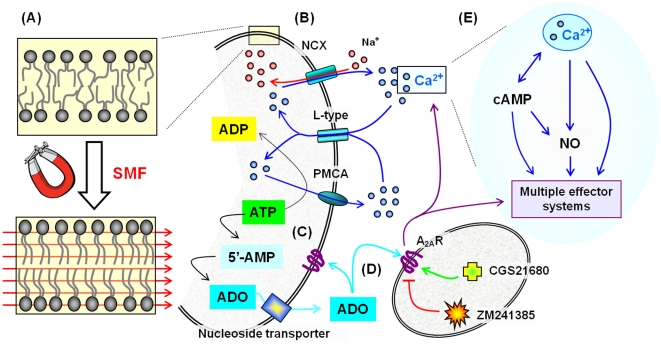
Outline of the putative mechanism of SMF on lipid bilayers, Ca^2+^ flux, A_2A_R receptors, and downstream modulation of multiple effector systems. (**A**) Phospholipid molecules possess diamagnetic anisotropy and align and reorient in the presence of moderate strength magnetic fields [60] thereby reducing the flexibility of the phospholipid acyl chains. The consequent stiffening of phospholipid molecules increases lateral compression and thickens the bilayer thereby altering the bulk biophysical properties of the membrane [79]. In turn, changes to membrane dynamics can affect the activity of embedded proteins ranging from signaling complexes (e.g., the toll like receptors [80]) to ion channels and membrane transporters [81]. (**B**) Specific examples of candidates for such SMF-mediated modulation include the sodium calcium exchanger (NCX) that transports Ca^2+^ out of a cell, the voltage gated L-type Ca^2+^ channel that transports Ca^2+^ into a cell, and the plasma membrane Ca^2+^ ATPase (PMCA) pump that hydrolyzes ATP to gain energy to remove Ca^2+^ from a cell (data relating to Ca^2+^ is given in Figure 1, concomitant changes to mRNA and proteins levels for A_2A_R in Figures 2 and 3, and ATP in Figure 4A). (**C**) ATP is linked to Ca^2+^ flux in another way, namely through metabolites such as adenosine (ADO, see Figure 4B-F). (**D**) ADO binds to adenosine receptors such as A_2A_R, which in turn can further modulate Ca^2+^ flux. (**E**) Calcium is a ubiquitous second messenger, leading to secondary responses that involve cAMP (Figure 5) or nitric oxide (Figure 6); in turn multiple effector systems (MES) can be engaged that affect additional endpoints including MAPK pathways (Figure 7), neurite outgrowth indicative of differentiation (Figure 8), and iron (Figure 9).

The biological effects of ZM241385 result from direct binding to A_2A_R [54]–[56]. By contrast, SMF – not being a conventional small molecule pharmacological agent – must elicit cellular responses through a fundamentally different mode of action. A plausible mechanism, consistent with the data shown in Figures 1–3 and outlined in cartoon form in Figure 10, is that SMF alters the biophysical properties of lipid bilayers [57]–[60], which in turn modulates ion channel activity [61] and Ca^2+^ levels [8], [21]. Over time periods of many hours to several days, SMF-initiated changes to Ca^2+^ can modulate signaling pathways, leading to significant changes in gene expression, cell behavior, and phenotype [8]. As a caveat, intracellular flux of calcium has not been thoroughly characterized in our experiments; for example, nuances of calcium release from storage organelles (e.g., the sarcoplasmic reticulum, which is affected by A_2A_R [62]) in SMF-treated cells remain largely undefined. In addition, calcium-initiated responses evoked by SMF may be augmented by calcium-independent mechanisms. For example, relevant to the endpoints measured in this study, activation of p42/p44 MAPK has been reported to mediate adenosine-induced nitric oxide production by both calcium-dependent and calcium-insensitive mechanisms [63]. Therefore we emphasize that although our results are fully consistent with a calcium-mediated mechanism, additional experiments are required to unequivocally establish ion channels as the “biosensor” that responds to magnetic exposure. Notwithstanding this ambiguity, SMF reproduced cellular effects of the A_2A_R antagonist AM241385 in multiple assays in PC12 cells in the current study. Together, these results raise the intriguing hypothesis that SMF can reproduce the effects of a promising class of non-dopaminergic PD drugs in a non-invasive manner and, more broadly, hold potential for ameliorating additional neurological disorders such as Alzheimer's and Huntington's diseases through modulation of A_2A_R [64]–[66].

## Materials and Methods

### Cell culture

Rat pheochromocytoma (PC12) cells were purchased from the American Tissue Culture Collection (ATCC, Manassas, VA) and grown in RPMI medium (GIBCO) that contained 10% horse serum, 5.0% fetal bovine serum, and penicillin/streptomycin (100 U/ml and 100 µg/ml, respectively). The cells were grown in a water saturated incubator maintained at 5.0% CO_2_ and 37°C and growth medium was changed twice a week. With the exception of the experiments evaluating neurite outgrowth (i.e., the results shown in Figure 8), the experiments described in this report used undifferentiated PC12 cells; this distinction is important because NGF-induced differentiated PC12 cells are less viable than undifferentiated cells [19] and undifferentiated PC12 cells have the capability to generate increased levels of cAMP (e.g., during early stages of anoxia) while NGF-induced PC12 cells have a diminished ability to produce cAMP [19].

### Exposure of cells to SMF

A problem hindering the acceptance of magnetic therapy has been that many studies have used inadequately defined treatment devices leading to difficulties reproducing experimental conditions from laboratory to laboratory [53]. Thus, in a previous publication [8] we carefully described our magnetic exposure conditions (using a device provided by the Advanced Magnetic Research Institute, International; AMRIi, Calgary, AB). Neodymium magnets are arranged in this device (shown at a publically available link: http://www.biomedcentral.com/imedia/1847435373293053/supp2.ppt) to provide a unidirectional field that varies between 0.23 and 0.28 T over several centimeters (≤1 mT/mm). Consequently, the shallow nature of this gradient ensures that individual cells are essentially exposed to uniform fields and are not subject to spatial gradient effects observed in cells exposed to fields that varied by ≥20 mT/mm [67]–[70]. Moreover, no dose-dependency was observed between 0.23 and 0.28 T for the endpoints tested in this report (data not shown). Untreated control cells were maintained in a separate (but otherwise identical) tissue culture incubator where the ambient magnetic field was measured at ∼52 mT, which is essentially identical to the 52,359 nT field reported for a latitude of 39° 19′ 35″ and a longitude of −76° 36′ 17″ (i.e., for United States zip code 21218) by the National Geophysical Data Center). Finally, we have tested whether orientation of the imposed field superimposed on – or opposing – the ambient geomagnetic field affects the endpoints being studied; field orientation was found to not have an effect (all experiments nonetheless were conducted using a superimposed field orientation).

### Measurement of Ca^2+^

To measure Ca^2+^, PC12 cells were grown in 12-well tissue culture plates for three days prior to the assay until they reached a confluency of 85 to 90%. For measuring extracellular Ca^2+^, PC12 cells were maintained in Ca^2+^ and Mg^2+^ free D-PBS (Dulbecco's phosphate buffered saline) for the indicated time intervals (e.g., as shown in Figure 1A) either with or without exposure to SMF. The supernatants were collected by centrifugation at 300 x *g* for 2.0 min and analyzed by using the Calcium Reagent Set (Pointe Scientific Inc., Canton, MI). For measuring intracellular Ca^2+^, the cells were lysed by sonication on ice for 1.0 min at an amplitude setting of 40 using a GE130PB ultrasonic processor (GE, New York, NY). In certain experiments, the optimized time point for evaluating Ca^2+^ efflux (i.e., 3.0 h) was used to evaluate the impact of A_2A_R agonist and antagonist on Ca^2+^ transport by pretreating the cells with 1.0 µM ZM241385 (4-(2-[7-amino-2-(2-furyl)[1], [2], [4]triazolo[2,3-a][1], [3], [5]triazin-5-ylamino]ethyl)-phenol, an adenosine A_2A_ receptor (A_2A_R)-specific antagonist, Tocris Bioscience, St. Louis, MO) or SMF for 40 min in Ca^2+^ and Mg^2+^ free D-PBS and then incubating each set of cells with 1.0 µM CGS21680 (4-[2-[[6-amino-9-(N-ethyl-b-D-ribofuranuronamidosyl)-9H-purin-2yl]amino]ethyl]benzenepropanoic acid hydrochloride, an adenosine A_2A_ receptor (A_2A_R)-specific agonist; Tocris Bioscience) or maintaining SMF exposure for an additional three hours before performing the assays described above.

### Quantitative real-time PCR (qRT-PCR) measurement of A_2A_R mRNA

PC12 cells were treated with L-type Ca^2+^ channel activator (10 nM Bay K8644) or L-type Ca^2+^ channel blocker (100 nM nifedipine) for 24 h; alternatively, they were incubated with CGS21680, ZM241385, or exposed to SMF as described above and used for qRT-PCR (described below) and western blot analysis (described in the following sections).

Forward and reverse primers for A_2A_R and the housekeeping gene glyceraldehyde-3-phosphate dehydrogenase (GAPDH) were designed by using Primer^3^ software [71] and were obtained from MWG-Biotech (High Point, NC). The sequences were as follows: A_2A_R 5′-GGACTCGGATTTGGATT-3′ (forward primer) and 5′-TGTTGGCAGCGTATGT-3′ (reverse primer); housekeeping genes glyceraldehyde-3-phosphate dehydrogenase (GAPDH): 5′-GCAAATTCCATGGCA CCGT-3′ (forward primer) and 5′-TCGCCCCACTTGATTTTGG-3′(reverse primer) were monitored in each experiment. The basic protocol followed for qRT-PCR experiments began with the isolation of total RNA from 5.0×10^6^ cells with the RNeasy Mini Kit (Qiagen, Valencia, CA) or by the TRIzol (Invitrogen) method. RNA quality was assessed by agarose gel electrophoresis (1.8% gels run with TAE buffer followed by nucleic acid band visualization under UV illumination after ethidium bromide staining) and quantified by *A*_260_/*A*_280_ OD readings. RNA integrity was confirmed using 18 S rRNA primers, and samples were standardized based on equal levels of β-actin cDNA. Quantitative real-time PCR was performed in an ABI Prism 7000 sequence detector (Applied Biosystems) using SYBR Green PCR Master Mix reagent (Applied Biosystems). Reactions were performed in 20 µl of a mixture containing a 2.0-µl cDNA dilution, 1.0 µl (10 pmol/μl) of each primer, and 10 µl of 2x SYBR master mix containing Amplitaq Gold DNA polymerase, reaction buffer, a dNTP mixture with dUTP, passive reference, and the SYBR Green I. PCR conditions were as follows: one cycle at 2.0 min at 50°C, then 95°C for 10 min, followed by 40 cycles of 95°C for 15 s and 60°C for 1.0 min. Specific PCR products were detected with the fluorescent double-stranded DNA binding dye, SYBR Green [72]. PCR amplification was performed in triplicate and replicated in three independent experiments. Gel electrophoresis and melting curve analyses were performed to confirm correct PCR product sizes and the absence of nonspecific bands. The expression levels of each gene were normalized against GAPDH using the comparative CT method according to the manufacturer's protocols [73].

### Plasma membrane preparation

PC12 cells were seeded in 100 mm culture dishes and pretreated with 1.0 µM CGS21680 for 30 min, then treated with 1.0 µM ZM241385 or exposed to SMF for 48 h. The cells were harvested by scraping from the plates and then collected by centrifugation at 300 x *g* for 2.0 min at 4.0°C. The plasma membrane protein extraction kit (BioVision, Mountain View, CA) was used according to the manufacturer's protocol to specifically isolate the plasma membrane from the total cellular membranes. The plasma membrane fraction was dissolved in 0.5% Triton X-100 in PBS, and plasma membrane protein concentration was measured using the BCA protein assay (Pierce) and then 40 µg protein of each sample was used for western blot analysis.

### Western blot analysis

An equal amount of protein from each sample (40 µg) was incubated for 5.0 min at 100°C in Laemmli buffer (Bio-Rad), separated on an 11% SDS-polyacrylamide discontinuous gel, and then electrophoretically transferred to a nitrocellulose membrane (Bio-Rad). The membrane was blocked with Tris-buffered saline containing 5.0% nonfat milk and 0.1% Tween 20 for 1.0 h at room temperature and then incubated overnight with rabbit phospho-p44/42 MAPK monoclonal antibody, p44/42 MAPK antibody (1∶1000 dilution) (Cell Signaling Technology, Beverly, MA) and anti-adensosine A_2A_ receptor rabbit antibody (Abcam Inc, Cambridge, MA) at 4.0°C, followed by anti-rabbit IgG, horseradish peroxidase-linked antibody (1∶2000) for 1.0 h. Bound antibody on the membrane was detected using the SuperSignal West Dura Extended Duration Substrate (Pierce) according to the protocols supplied by the manufacturer. Quantification of bands was performed by using the NIH ImageJ software (available on the World Wide Web at rsb.info.nih.gov/nih-image) following a published method [74].

### Measurement of cellular ATP

ATP was measured using a chemiluminescence method employing a luciferin–luciferase reaction [75]; the assay reagents were purchased as a kit (ATP bioluminescent somatic cell assay kit, FL-ASC; Sigma) and prepared according to the manufacturer's instructions. PC12 cells were plated on 35-mm-diameter dishes and left untreated, incubated with 1.0 µM or CGS21680 or 1.0 µM ZM241385, or exposed to SMF for 6.0 h cells and then harvested and suspended in 0.5 ml of RPMI. Cell samples (50 µl) were placed into a tube that contained 100 µl of somatic cell releasing reagent and 50 µl of sterile purified water (SAM) or an ATP standard (2.0 nmol/ml) as internal standard (IS) and swirled briskly. An portion of this mixture (100 µl) was transferred to a reaction vial that contained 100 µl of assay mix solution, and then the amount of light emitted, *L*, was immediately measured with a luminometer (Modulus, Turner Biosystem, Sunnyvale, CA). The amount of ATP in the cell sample was calculated by the following equation: *ATP*S_AM_  =  *ATP*_IS_ X *L*_SAM_/(*L*_SAM+IS_-*L*_SAM_), where *ATP*_SAM_ stands for the ATP in the cell sample, *ATP*_IS_ for the ATP in the internal standard, *L*_SAM_ for the light emitted by the cell sample, and *L*_SAM+IS_ for the light emitted by the cell sample plus the internal standard.

### HPLC measurement of adenosine (ADO) levels

PC12 cells were plated in 35 mm tissue culture dishes. When the cells reached 90% confluency, the dishes were rinsed twice with 2.0 ml of Krebs solution that contained 135 mM NaCl,1.5 mM NaH_2_PO_4_, 5.0 mM KCl, 2.0 mM CaCl_2_, 2.0 mM MgCl_2_, 10 mM glucose, 15 mM HEPES, and the ADO deaminase (ADA) inhibitor [erythro-9-(2-hydroxy-3-nonyl)adenosine (EHNA) at 1.0 µM. To measure the release of ADO using a procedure that avoids the potential production of ADO via the degradation of endogenous adenine nucleotides, 0.5 ml of the prewarmed Krebs solution including 1.0 µM EHNA was placed onto the plated cells (EHNA was included to inhibit extracellular degradation of ADO by ADA). After incubation for 3.0 h, the extracellular fluid was collected and the samples were processed to extract ADO as reported previously [28], [76]; briefly, the extracellular fluids were rapidly collected into microcentrifuge tubes and centrifuged at 14,000 x *g* for 1.0 min. Supernatants (400 µl) were placed into separate tubes and deproteinated with 20 µl of 100% trichloroacetic acid. The acid-precipitated protein was removed by centrifugation at 14,000 x *g* for 5.0 min, and 300 µl of supernatant was immediately neutralized with 40 µl of 3.3 M KOH. The adenine nucleotides were precipitated by adding 200 µl of 1.0 M zinc sulfate and 100 µl of saturated barium hydroxide, vortex mixing for 10 s, and centrifuging at 14,000 x *g* for 5.0 min. The samples were then analyzed by HPLC; separation of compounds was achieved using a 4-mm (i.d.), 15-cm-long prepacked Novapak C18 column. Samples were eluted from the column using a gradient (0–40%, 35 min) of low-strength eluent (0.02 mol/L potassium dihydrogen phosphate buffer, pH 5.5) and high-strength eluent [60∶40 (vol/vol) mixture of methanol and water]. The flow rate was 1.0 ml/min, and the column temperature was ambient in all the determinations. Peaks were identified on the basis of retention times in comparison with an authentic standard of ADO.

### cAMP assays

On the day of the cAMP assay, 5.0×10^5^ cells were placed in fresh medium and incubated with 1.0 µM CGS21680, 1.0 µM ZM241385, or SMF in the presence of 1.0 U/ml adenosine deaminase (ADA) for 3.0 h. Cells were then harvested, lysed in 500 µl 0.1 M HCl for 20 min, centrifuged at 700 *x g* for 10 min, and the supernatants were assayed for cellular cAMP accumulation using the cAMP enzyme immunoassay system kit (Sigma-Aldrich, St. Louis, MO) following protocols supplied by the manufacturer.

### Nitrite assays

Nitric oxide (NO) production was assessed by measuring the amount of nitrite, a stable metabolic product of NO that provides an indirect measurement of NO, by using the Griess diazotization reaction [77]. Briefly, after 24 h after incubation with 1.0 µM CG21680 or 1.0 µM ZM241385 or exposure to SMF, samples of medium (150 µl) were collected from cells and mixed with 130 µl dH2O and with 20 µl Griess reagent using instruction supplied by the manufacturer (Cat. No. G-7921, Invitrogen-Molecular Probes, Carlsbad, CA). After a 30 min incubation period at room temperature, the samples were evaluated spectrophotometrically at 548 nm and OD values – in comparison with a standard curve was determined in culture medium by using serial dilutions of sodium nitrite – represented total stable metabolites of NO.

### Measurement of neurite outgrowth

PC12 cells grown on coverslips were changed into differentiation medium (1.0% horse serum with 25 ng/ml NGF) 24 h after being passaged. The cells were then pretreated with 1.0 µM CGS21680 for 30 min followed by the addition of 1.0 µM ZM241385 or exposure to SMF for an additional three days followed by staining with F-actin conjugated with Oregon Green488 phalloidin (1∶100) (Molecular Probes, now Invitrogen, Eugene, OR). The coverslips were mounted using ProLong Gold® anti-fade reagent (Molecular Probes, Cat. No. P36934) and imaged by using a Zeiss 510 Meta confocal microscope. From each slide at least 100 cells from five randomly selected fields were counted. Cells were classed as differentiated if they exhibited an outgrowth extending from the cell which was at least 1.5 times the diameter of the cell. Measurements were carried out using NIH Scion image software.

### Quantification of intracellular iron

Intracellular iron was quantified using a colorimetric assay described by Riemer et al [78]. Briefly, stimulus-treated or control PC12 cells grown in 48-multiwell cell culture plates were incubated in the presence of 50 µM FeSO_4_ for 2.0 h. The culture medium was removed and cells were washed twice with ice-cold PBS. Cells were frozen in the culture plates and stored at −20°C. Cells were lysed with 50 mM NaOH for 2.0 h on a shaker in a humidified atmosphere. Aliquots of these samples were incubated with equal amounts of 10 mM HCl and a 1∶1 solution of 1.4 M HCl/4.5% (wt/vol) KMnO_4_ for 2.0 h in a 60°C prewarmed water bath under the fume hood to release all intracellular protein-bound iron. The cells were allowed to cool to room temperature before 60 µl of a 60°C detection solution containing 6.5 mM ferrozine (Sigma), 6.5 mM neocuproine (Sigma), 2.5 M ammonium acetate, and 1.0 M ascorbic acid (Sigma) was added. Color reading of the supernatant was done in an ELISA reader at 550 nm. For quantification, an appropriate standard curve was prepared by using a 10 mM FeSO_4_ stock solution.

### Statistical analysis

Results are expressed as mean values of three or more independent experiments and error bars represent standard error of the mean (S.E.M.) calculations; statistical analyses were made using the Student unpaired *t*-test or ANOVA followed by the appropriate post hoc tests.
